# The impact of a multidisciplinary team on the management of fracture-related infections: A surveillance study in Brazil

**DOI:** 10.1016/j.bjid.2025.104576

**Published:** 2025-09-11

**Authors:** Icaro Santos Oliveira, Carlos Augusto Finelli, Taiana Cunha Ribeiro, Carolina Coelho Cunha, Thomas Stravinskas Durigon, Rodrigo Peixoto Vargas, Rafael Brull Tuma, Giselle Burlamaqui Klautau, Fernando Baldy dos Reis, Mauro Jose Salles

**Affiliations:** a Universidade Federal de São Paulo (UNIFESP), Escola Paulista de Medicina (EPM), Grupo de Infecção Musculoesquelética (Ortoinfecto), Departamento de Ortopedia e Traumatologia, São Paulo, SP, Brazil; b Faculdade de Ciências Médicas da Santa Casa de São Paulo, Disciplina de Infectologia, São Paulo, SP, Brazil; c Universidade Federal de São Paulo (UNIFESP), Escola Paulista de Medicina (EPM), Laboratório Especial de Microbiologia Clínica (LEMC), Divisão de Doenças Infecciosas, Departamento de Medicina, São Paulo, SP, Brazil

**Keywords:** Fracture-related infection, Multidisciplinary teams, Biofilm, Osteomyelitis, Antibiotic stewardship, Prevention, Diagnosis

## Abstract

**Background:**

Fracture-Related Infection (FRI) is an increasing and challenging complication following orthopedic trauma surgery. Preventive and microbial diagnostic measures vary significantly particularly in low- and middle-income countries. The objectives of this national questionnaire were to investigate clinical practices towards preventive and diagnostic strategies adopted by Brazilian orthopedic trauma centers and to assess the impact of Multidisciplinary Teams (MDT) on the management of FRI.

**Methods:**

A 34-item electronic questionnaire was developed via REDCap® and distributed to all trauma surgeons registered of the Brazilian Society of Orthopedics and Traumatology (SBOT).

**Results:**

With a response rate of 24 %, the survey was fully responded by 140 trauma surgeons, 63.6 % of them working in southeast region centers. Collaborative work with MDT focused on musculoskeletal infections was reported by only 41.0 %. Cephalosporins were universally prescribed as Perioperative Antibiotic Prophylaxis (PAP), while association with an aminoglycoside increased (35.0 %) for severe open fracture. One-day duration of PAP for closed fracture was prescribed in 68.1 %, while it often exceeded current recommendations. Diagnostic practices for FRI patients were primarily based on clinical signs and standard radiological and laboratory tests, with limited use of microbiological techniques. Trauma services working collaboratively with MDT significantly improved FRI management, including, use of sonication fluid for diagnosis (46.6 % vs. 26.8 %; *p* = 0.02), body weight-adjusted antibiotic dosing for PAP (50.0 % vs. 24.4 %; *p* = 0.02), appropriate duration of PAP according to the severity of soft-tissue damage (80.7 % vs. 59.3 %; *p* = 0.01), infection risk stratification in elderly patients with fractures (45.6 % vs. 21.0 %; *p* < 0.001), use of negative-pressure wound therapy (87.9 % vs. 54.9 %; *p* < 0.001) and regular collaboration with orthoplastic surgeon (44.8 % vs. 17.5 %; *p* = 0.01).

**Conclusions:**

This national survey revealed marked heterogeneity in FRI management across Brazilian trauma services. Ongoing MDT collaboration improved clinical practice, especially diagnostic work-up and antimicrobial stewardship.

## Introduction

Fracture-Related Infection (FRI) represents the most serious and feared orthopedic complication associated with musculoskeletal trauma.^1^ Globally, FRI rates range from approximately 1 % in closed fractures to up to 30 % in open fractures.^2^ In Low- and Middle-Income Countries (LMICs), where traffic-related trauma remains a major public health issue, infection rates in complex lower limb open fractures range from 13.9 % to 23 %.^3^ In 2020 alone, over 190,000 hospitalizations and 32,716 deaths were attributed to Road Traffic Accidents (RTA), with motorcyclists accounting for 61.6 % of hospitalizations and 36.7 % of fatalities ‒ mostly affecting individuals under 40 years of age.^4^ Consequently, the estimated annual economic burden of RTA in Brazil reaches approximately BRL 50 billion, excluding productivity losses commonly measured in Disability-Adjusted Life Years (DALYs).^5^^,^^6^ Within this national context, FRI rate is estimated at around 15.9 %.^7^ These infections are frequently polymicrobial and often require prolonged antimicrobial therapy and repeated surgical interventions, with severe consequences including limb amputation, contributing significantly to the financial burden on healthcare systems.^8^ FRI management has been associated with a 2.5-fold increase in surgical and hospitalization costs, in addition to substantial spending on broad-spectrum antimicrobials used to treat these infections.^9^

Given the complexity and heterogeneity of patients undergoing treatment for FRI, the involvement of a Multidisciplinary Team (MDT) has emerged as a promising strategy to improve clinical outcomes.^10^ Typically composed of orthopedic trauma and plastic surgeons, infectious disease specialists, microbiologists, and clinical pharmacists, MDTs operate across the pre-, peri‑, and postoperative periods.^11^ Their role includes ensuring adherence to evidence-based preventive measures and to antimicrobial stewardship, microbial diagnostic accuracy, and adequate surgical decision-making. Ultimately, their involvement has proved to mitigate rates of revision surgery, decreased amputation rates, and improved quality of life for affected patients.^12^ Positive experiences have been documented in the literature regarding MDT interventions in Musculoskeletal Infections (MSIs), particularly with respect to optimizing antimicrobial use, reducing the emergence of Multidrug-Resistant (MDR) organisms, and preventing adverse events such as *Clostridioides* difficile infection.^13^ For example, a German institution demonstrated that weekly MDT meetings to manage periprosthetic joint infections and spondylodiscitis were associated with reduced antimicrobial use, shorter hospital stays, and lower rates of revision procedures.^14^^,^^15^ Similarly, the MDT approach was beneficial in managing complex extremity defects, often avoiding unnecessary limb amputations.^16^

The formal implementation of MDTs specifically focused on FRI management is a relatively recent development. Until 2018, there was no universally accepted definition of FRI.^17^ That year, an international expert panel established consensus diagnostic criteria based on existing knowledge from periprosthetic joint infections.^18^ Despite this advancement, considerable heterogeneity persists in clinical practice, including variability in the number of intraoperative tissue samples, transport and processing protocols, and the diagnostic use of inflammatory markers, imaging, molecular diagnostics, sonication fluid cultures, and histopathology.^19^, ^20^, ^21^ These inconsistencies contribute to underdiagnosis and inappropriate treatment, often resulting in severe, irreversible complications. In Brazil, the situation is further aggravated by the country's continental dimensions, regional disparities in socioeconomic development, and unequal access to healthcare services, both public and private. These factors lead to marked variation in hospital infrastructure, medical technology, implant availability, and the presence of trained personnel to manage FRI cases.^8^ Additionally, the national landscape is characterized by a lack of epidemiological and microbiological data, inconsistent adoption of preventive protocols, and the absence of standardized diagnostic tools for FRI.^7^

Considering these challenges, MDT implementation tailored to FRI management emerges as a potentially impactful strategy in Brazil. However, limited data are available regarding global FRI rates and the adoption of standardized preventive and diagnostic practices, particularly in LMICs. To address these gaps, we conducted a nationwide survey to characterize the current practices of orthopedic trauma surgeons in Brazil. Our objectives were to assess the national scenario and to evaluate the potential impact of MTD on the management of these complex infections.

## Material and methods

### Questionnaire development

This study was conducted as a survey, in which the questionnaire was developed in August 2022 by a consolidate MDT at a tertiary academic university hospital. The items for the epidemiological survey were designed using the modified Delphi method.^22^ This prospective, qualitative approach aims to achieve consensus among experts on a given subject through iterative rounds of discussion, participant anonymity, and structured feedback. Each round allows for the refinement of hypotheses and expert judgments until consensus or response stability is attained. For the purposes of this study, a policy Delphi approach was applied, conducted in the format of a Mini Delphi.^23^^,^^24^ The first round consisted of a meeting with approximately twenty orthopedic trauma specialists at the Orthopaedic Department of the university hospital. During this session, the study’s aims were presented, and ten broad, open-ended questions related to the topic were posed (Appendix A). Based on the responses obtained, two subsequent rounds of discussion were held, leading to the development of a more refined and specific version of the questionnaire. The final version was structured according to the framework proposed by Marques, JBV et al.^25^ The study was conducted in accordance with the World Medical Association Code of Ethics (Declaration of Helsinki) for research involving human subjects. The study protocol was approved by the local Research Ethics Committee under protocol number CAAE: 65,457,622.2.1001.5505 n° 6.095.500.

### Questionnaire structure

The final questionnaire (Appendix B) comprised thirty-four questions divided into three sections: 1) Demographic data; 2) Strategies for FRI prevention; and 3) FRI diagnosis. The prevention section was further divided into four subsections: a) MDT involvement; b) Irrigation and debridement practices; c) Antimicrobial prophylaxis; and d) Skin and soft tissue management. All questions were formulated to be objective, concise, and free of ambiguous language.^26^ Two types of multiple-choice questions were included: some required participants to choose a single best answer from a predefined list, while others presented affirmative statements evaluated using a Likert scale ranging from “never” to “always”.^27^ In some instances, participants were allowed to select more than one answer. A brief introductory text was provided alongside the questionnaire, clearly outlining the study’s objectives and emphasizing the importance of participant contributions. The voluntary nature of participation was highlighted, and the estimated completion time for the survey was approximately ten minutes. Participants who agreed to participate provided informed consent and retained a copy of the consent document.^28^ A pilot test was carried out during the National Congress on Trauma Surgery in November 2022, in which, the questionnaire was distributed to trauma specialists to evaluate the tool’s relevance, feasibility, redundancy, reliability, and validity.^29^

### Questionnaire distribution

The final version of the questionnaire was administered via the REDCap® platform and distributed by email in August 2023 to all orthopedic trauma surgeons registered with the Brazilian Society of Orthopedics and Traumatology (SBOT). The survey remained open for one month. Non-respondents received up to two reminder emails between two and four weeks following the initial invitation, and the survey was additionally promoted through a QR code. SBOT is a national professional society dedicated to the advancement of orthopedic knowledge and the dissemination of evidence-based practices in musculoskeletal trauma care.

### Statistical analysis

Descriptive statistics were applied to analyze all collected data. Categorical variables were presented as absolute frequencies and percentages. The survey response rate was calculated by dividing the number of completed questionnaires by the total number of invitations sent. To assess whether consistent collaboration with a fully MDT was associated with more structured practices in the prevention and diagnosis of FRIs, a subgroup analysis was performed. Respondents were classified into two groups based on their reported frequency of MDT collaboration: the “with MDT” group included those who answered “always” on the Likert scale, while the “without MDT” group comprised those who selected “frequently”, “occasionally”, “rarely”, or “never”. This binary classification was chosen to distinguish consistently structured MDT environments from those with less formal or variable collaboration. It aimed to reflect the variability in MDT implementation observed across Brazil and to explore the potential impact of more robust, institutionalized team structures. For the purposes of this study, an MDT dedicated to the management of musculoskeletal infections was operationally defined as a structured team consisting of an orthopaedic surgeon, an infectious disease specialist, a clinical pharmacist, and a specialized nurse. Depending on complex cases management, other specialists such as plastic surgeons could also be involved. This definition aligns with the framework proposed by Vasoo et al., who advocates for multidisciplinary, guideline-driven approaches to bone and joint infections.^10^ Bivariate analyses between MDT status and other variables were performed using the Chi-Square test or Fisher’s exact test, as appropriate. Statistical significance was set at a p-value < 0.05, with a 95 % Confidence Interval.

## Results

### Participant demographics

The survey was distributed via email to 587 orthopedic trauma surgeons registered with SBOT in 2023. A total of 143 participants accessed the form, and 140 completed the questionnaire in full, yielding a response rate of 24 %. Most respondents practice in the Southeast region of Brazil (*n* = 89; 63.6 %), where they more frequently collaborate with multidisciplinary teams (*p* = 0.06), with a relatively balanced distribution between private (*n* = 40; 28.6 %) and public healthcare institutions (*n* = 35; 25.0 %). A significant proportion reported having over ten years of professional experience (*n* = 79; 56.5 %). Nonetheless, only 41.4 % of respondents indicated consistent collaboration with the MDT at their institutions for the management of FRIs (Table 1).

**Table 1:** Regional distribution of participants' demographic characteristics.

	**Total, n (%)**	**North, n (%)**	**Northeast, n (%)**	**Midwest, n (%)**	**Southeast, n (%)**	**South, n (%)**	**p-value**
	**140 (100)**	**3 (2.1)**	**18 (12.9)**	**6 (4.3)**	**89 (63.6)**	**24 (17.1)**	
**Practice setting**							
Public	35 (25.0)	1 (33.3)	6 (33.3)	2 (33.3)	23 (25.8)	3 (12.5)	0.69
Private	40 (28.6)	1 (33.3)	5 (27.8)	4 (66.7)	27 (30.3)	3 (12.5)	0.25
Mixed	30 (21.4)	0 (0.0)	3 (16.7)	0 (0.0)	20 (22.5)	7 (29.2)	0.59
Philanthropic	10 (7.1)	0 (0.0)	2 (11.1)	0 (0.0)	3 (3.4)	5 (20.8)	0.06
Academic	21 (15.0)	0 (0.0)	2 (11.1)	0 (0.0)	14 (15.7)	5 (20.8)	0.72
Not reported	4 (2.9)	1 (33.3)	0 (0.0)	0 (0.0)	2 (2.2)	1 (4.2)	0.03
**Training duration**							
0 – 5 years	32 (22.9)	0 (0.0)	1 (5.6)	1 (16.7)	19 (21.3)	11 (45.8)	0.07
5 – 10 years	24 (17.1)	0 (0.0)	8 (44.4)	1 (16.7)	12 (13.5)	3 (12.5)	0.05
10 – 20 years	40 (28.6)	2 (66.7)	5 (27.8)	2 (33.3)	26 (29.2)	5 (20.8)	0.72
> 20 years	39 (27.9)	1 (33.3)	4 (22.2)	2 (33.3)	28 (31.5)	4 (16.7)	0.77
Not reported	5 (3.6)	0 (0.0)	0 (0.0)	0 (0.0)	4 (4.5)	1 (4.2)	0.88
**MDT teamwork**							
Always	58 (41.4)	0 (0.0)	4 (22.2)	3 (50.0)	44 (49.4)	7 (29.2)	0.27
Frequently	43 (30.7)	2 (66.7)	5 (27.8)	0 (0.0)	32 (36.0)	4 (16.7)	0.24
Occasionally	24 (17.1)	1 (33.3)	5 (27.8)	2 (33.3)	8 (9.0)	8 (33.3)	0.05
Rarely	7 (5.0)	0 (0.0)	2 (11.1)	1 (16.7)	2 (2.2)	2 (8.3)	0.29
Never	8 (5.7)	0 (0)	2 (11.1)	0 (0.0)	3 (3.4)	3 (12.5)	0.38

MDT, Multidisciplinary Team.

### Prevention measures

In the initial management of open fractures, most surgeons reported performing irrigation with normal saline without additives (*n* = 117; 83.6 %), typically using free pressure techniques (*n* = 111; 79.3 %). The irrigation volume was adjusted according to fracture severity: 3 to 6 liters for Gustilo-Anderson type I or II fractures (*n* = 68; 48.6 %) and 9 liters or more for type III fractures (*n* = 76; 54.3 %). Debridement of devitalized tissue was generally performed within six hours after trauma, both for type GI or GII fractures (*n* = 78; 55.7 %) and for type GIII fractures (*n* = 87; 62.1 %). In cases of complex trauma with severe soft tissue damage, only 28.6 % of respondents reported regular collaboration with an orthoplastic team. However, negative pressure wound therapy was widely used as a preventive measure against FRIs (*n* = 96; 68.6 %) (Table 2).

**Table 2:** Preventive strategies in the management of fracture-related infections.

	**Total, n (%)**
	**140 (100.0)**
**Irrigation solution type for open fractures**	
Normal saline	117 (83.6)
Normal saline + antibiotics	6 (4.3)
Normal saline + antiseptics	16 (11.4)
Not reported	1 (0.7)
**Irrigation pressure for open fractures**	
Without pressure	111 (79.3)
Low pressure	21 (15)
High pressure	6 (4.3)
Not reported	2 (1.4)
**Saline volume for irrigation of GI or GII**	
< 3L	10 (7.1)
3 – 6L	68 (48.6)
7 – 9L	26 (18.6)
> 9L	35 (25.0)
Not reported	1 (0.7)
**Saline volume for irrigation of GIII**	
3 – 6L	27 (19.3)
7 – 9L	37 (26.4)
> 9L	76 (54.3)
**Average time from trauma to debridement of GI or GII**	
< 6h	78 (55.7)
6 – 24h	59 (42.1)
24 – 48h	2 (1.4)
> 48h	1 (0.7)
**Average time from trauma to debridement of GIII**	
< 6h	87 (62.1)
6 ‒ 24h	48 (34.3)
24 – 48h	1 (0.7)
> 48h	1 (0.7)
Not reported	3 (2.1)
**Average time from trauma to PAP in GI or GII**	
< 3h	112 (80.0)
3 – 6h	20 (14.3)
7 – 12h	5 (3.6)
13 – 24h	2 (1.4)
Not reported	1 (0.7)
**Average time from trauma to PAP in GIII**	
< 3h	120 (85.7)
3 ‒ 6h	14 (10.0)
7 ‒ 12h	3 (2.1)
13 ‒ 24h	2 (1.4)
Not reported	1 (0.7)
**Collaboration with orthoplastics**	
Always	40 (28.6)
Frequently	40 (28.6)
Occasionally	26 (18.6)
Rarely	17 (12.1)
Never	15 (10.7)
Not reported	2 (1.4)
**Use of negative pressure therapy**	
Yes	96 (68.6)
No	44 (31.4)

GI, Gustilo-Anderson type I open fracture; GII, Gustilo-Anderson type II open fracture; GIII, Gustilo-Anderson type III open fracture; PAP, Perioperative Antibiotic Prophylaxis.

### Antimicrobial prophylaxis

**Fig. 1:**
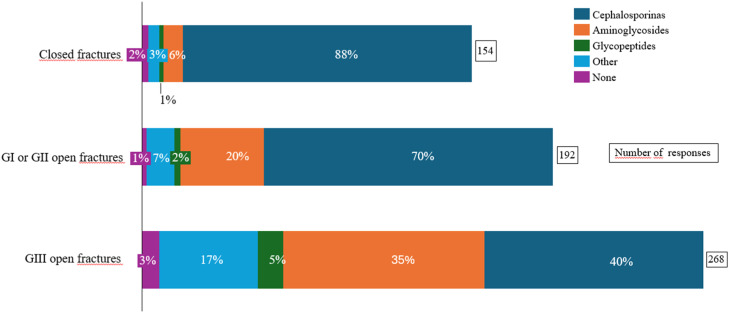
Type of perioperative antibiotic prophylaxis used in fracture management.

Surgical antimicrobial prophylaxis for open fractures was administered within three hours after injury in most cases, for both types of GI or GII fractures (*n* = 112; 80.0 %) and type GIII fractures (*n* = 120; 85.7 %) (Table 2). Cephalosporins were the most frequently reported antibiotics used for prophylaxis in closed fractures (*n* = 135; 88 %), as well as in type GI or GII (*n* = 135; 70.0 %) and type GIII (*n* = 108; 40.0 %) open fractures. The use of aminoglycosides increased with the risk of contamination, particularly in the management of type GIII open fractures (*n* = 94; 35.0 %). Other regimens were also reported (Fig. 1).

**Fig. 2:**
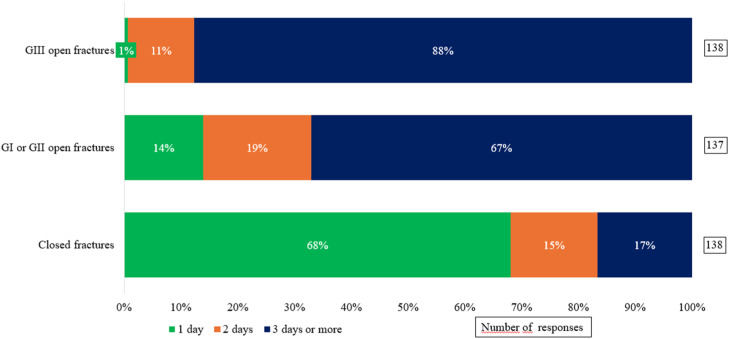
Duration of perioperative antibiotic prophylaxis in fracture management.

Regarding the duration of prophylaxis, antibiotics were administered for one day in 94 cases (68.0 %) of closed fractures. For type GI or GII open fractures, 92 respondents (67.0 %) reported maintaining antimicrobial prophylaxis for three or more days, a practice also followed by 121 respondents (88.0 %) in cases of type GIII fractures (Fig. 2).

Only 49 respondents (35.0 %) routinely adjusted antibiotic dosages based on patient body weight, whereas 16 (11.4 %) reported never performing this adjustment. Additionally, infection risk stratification in elderly patients with intertrochanteric fractures to guide the duration of surgical prophylaxis was consistently applied by only 16 respondents (11.6 %), while 53 (38.4 %) reported never employing this approach. Local antibiotic therapy for open fractures was reported as very rarely used by 33 respondents (23.9 %) for type GI–II fractures and by 44 (31.7 %) for type GIII fractures. When local therapy was used, Polymethylmethacrylate (PMMA) was the most frequently reported carrier (*n* = 23; 16.4 %). At hospital discharge, antimicrobial prescriptions were commonly issued regardless of the initial contamination risk, with cephalexin being the most frequently prescribed oral antibiotic (*n* = 69; 49.3 %).

### Diagnosis

**Fig. 3:**
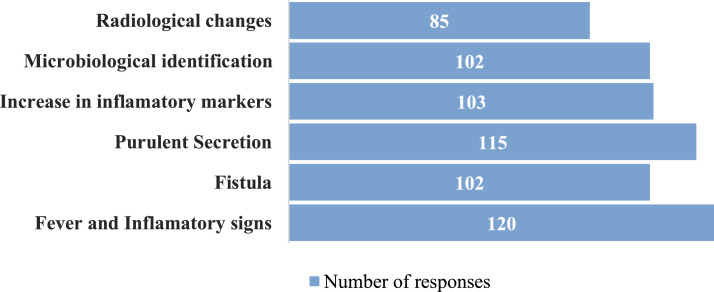
Diagnostic parameters of FRIs.

When asked about diagnostic practices for FRIs, respondents reported relying primarily on clinical signs and symptoms (e.g., fever and local inflammatory signs [*n* = 120; 85.7 %]), blood tests [elevated inflammatory markers (*n* = 103; 73.6 %)], and radiographic findings (*n* = 85; 60.7 %). Despite current international recommendations, microbiological identification methods were reported less frequently (*n* = 102; 72.9 %) (Fig. 3). During surgical procedures for suspected FRI, three or more tissue samples were collected intraoperatively in 122; 87.8 % of cases, with bone fragments (*n* = 120; 85.7 %) and deep tissue specimens (*n* = 118; 84.3 %) being the most frequently sampled. Sonication fluid analysis was performed by only 49 respondents (35.0 %), and histopathological analysis was rarely used (*n* = 56; 40.3 %).

### Impact of collaboration with MDT

Trauma services routinely collaborating with MDT, containing both orthopedic trauma and infectious disease specialists, demonstrated improved implementation of both prophylactic and diagnostic strategies for FRI management. These institutions reported significantly higher adoption of the following practices: use of sonication fluid for diagnostic purposes (46.6 % vs. 26.8 %; *p* = 0.02), weight-adjusted antibiotic dosing (50.0 % vs. 24.4 %; *p* = 0.02), appropriate duration of prophylaxis according to fracture type (80.7 % vs. 59.3 %; *p* = 0.01), guidance on local antibiotic therapy (37.9 % vs. 16.0 %; *p* = 0.003), infection risk stratification in elderly fracture patients (45.6 % vs. 21.0 %; *p* < 0.001), use of negative pressure wound therapy (87.9 % vs. 54.9 %; *p* < 0.001), and regular collaboration with orthoplastic teams (44.8 % vs. 17.5 %; *p* = 0.01) (Table 3).

**Table 3:** Relationship between preventive and diagnostic approaches in fracture-related infection management and multidisciplinary collaboration.

	**Without MDT**		**With MDT**		
	**n (%)**	**95% CI [%]**	**n (%)**	**95% CI [%]**	**p-value**
**Weight-based antibiotic dosing for PAP**					
Always	20 (24.4)	[15.1‒33.7]	29 (50.0)	[37.1‒62.9]	0.02
Frequently	17 (20.7)	[11.9‒29.5]	7 (12.1)	[3.7‒20.5]	
Occasionally	18 (22.0)	[13.0‒31.0]	10 (17.2)	[7.5‒26.9]	
Rarely	14 (17.1)	[9.0‒25.2]	9 (15.5)	[6.2‒24.8]	
Never	13 (15.9)	[8.0‒23.8]	3 (5.2)	[0.0‒10.9]	
**Length of PAP in the management of closed fractures**					
1 day	48 (59.3)	[48.6‒70.0]	46 (80.7)	[70.5‒90.9]	0.01
2 days or more	33 (40.7)	[30.0‒51.4]	11 (19.3)	[9.1‒29.5]	
**Carrier-free local therapy for open fracture management**					
Always	2 (2.5)	[0.0‒5.9]	0 (0.0)	[0.0‒1.7]	
Frequently	4 (4.9)	[0.0‒10.4]	3 (5.2)	[0.0‒11.9]	
Occasionally	6 (7.4)	[1.7‒13.1]	10 (17.2)	[7.5‒26.9]	
Rarely	13 (16.0)	[8.0‒24.0]	22 (37.9)	[25.4‒50.4]	
Never	56 (69.1)	[59.0‒79.2]	23 (39.7)	[27.1‒52.3]	0.003
**Risk assessment for guiding preemptive therapy in elderly fracture patients**					
Always / Frequently	17 (21.0)	[12.1‒29.9]	26 (45.6)	[32.7‒58.5]	<0.001
Occasionally	8 (9.9)	[3.4‒16.4]	11 (19.3)	[9.1‒29.5]	
Rarely / Never	56 (69.1)	[59.0‒79.2]	20 (35.1)	[22.7‒47.5]	
**Collaboration with orthoplastic team**					
Always	14 (17.5)	[9.2‒25.8]	26 (44.8)	[32.0‒57.6]	0.01
Frequently	29 (36.3)	[25.8‒46.8]	11 (19.0)	[8.9‒29.1]	
Occasionally	15 (18.8)	[10.2‒27.4]	11 (19.0)	[8.9‒29.1]	
Rarely	11 (13.8)	[6.2‒21.4]	6 (10.3)	[2.5‒18.1]	
Never	11 (13.8)	[6.2‒21.4]	4 (6.9)	[0.0‒14.6]	
**NPWT for managing skin and soft tissue injuries**					
Yes	45 (54.9)	[44.1‒65.7]	51 (87.9)	[80.8‒95.0]	<0.001
No	37 (45.1)	[34.3‒55.9]	7 (12.1)	[5.0‒19.2]	
**Diagnosis of FRI using sonication fluid culture**					
Yes	22 (26.8)	[63.6‒82.8]	27 (46.6)	[33.8‒59.4]	0.02
No	60 (73.2)	[17.2‒36.4]	31 (53.4)	[40.6‒66.2]	

FRI, Fracture-Related Infection; MDT, Multidisciplinary Team; PAP, Perioperative Antibiotic Prophylaxis; NPWT, Negative Pressure Wound Therapy.

## Discussion

In agreement with previous experiences, the findings of this study demonstrate that orthopedic trauma services working alongside with MDTs improve the management of FRIs.^14^^,^^15^ Improvements were observed in microbial diagnostic yield, adjustment of prophylactic antimicrobial dosing based on patient weight, tailored antibiotic duration according to fracture severity, guidance on local antimicrobial therapy, and infection risk stratification in elderly patients with fractures. Worryingly, this study highlights the limited availability of MDTs advising the FRI management in Brazil. The lack of a multidisciplinary vision encompassing infectious diseases and microbiologists’ specialists has been associated with poor clinical outcomes, including severe complications such as amputations.^8^ Conversely, Hanssen et al. reported that adherence to multidisciplinary decision-making in MSI management at a tertiary academic center was associated with improved outcomes, whereas the lack of such collaboration correlated with reduced treatment success.^30^

In terms of preventive strategies, our findings revealed heterogeneity in antibiotic prophylaxis practices for both closed and open fractures. Although cephalosporins and aminoglycosides were the most prescribed agents, other antimicrobials (vancomycin, clindamycin) were also prescribed. However, the administration of these drugs frequently deviated from international guidelines,^31^ potentially contributing to the high prevalence of antimicrobial resistance among Brazilians hospital settings. Integrating an antimicrobial stewardship program within MDT ‒ comprising clinical microbiology and pharmacy professional – may assist to address this issue, as previously demonstrated by Vidal et al.^32^ The duration of prophylactic antibiotic therapy also varied widely. Shorter courses were significantly common for closed fractures in settings counting with MDT. Among those hospitals lacking MDT support, open fractures received extended prophylaxis, especially in more severe cases. These findings diverge from international recommendations, which advocate limited perioperative antibiotic use, usually not exceeding 72 h, even in severe open fractures as reported in a systematic review[32] and supported by Vanvelk et al., who found no benefit from prolonged prophylaxis in preventing FRIs.^33^

Only a minority of surveyed surgeons consistently adjusted the antibiotic dosing based on patient`s body weight ‒ a practice more significantly commonly observed in institutions counting with MDT. The positive influence of these teams on optimizing antimicrobial prescriptions has been well documented, as illustrated by Bauer et al., who emphasized improvements in dosing adjustments based on renal function and body weight.^34^ More recently, Royere et al. reinforced the essential role of pharmacy professionals in dosing optimization, drug interaction management, and minimizing antimicrobial-related adverse events within MDTs.^35^

Local antimicrobial therapy has increasingly been recognized for its potential to enhance antibiotic concentrations at the site of trauma and reduce the risk of osteomyelitis.^36^ This approach was effective in a randomized clinical trial where local vancomycin powder significantly decreased infection rates caused by gram-positive organisms in open fractures.^37^ Nevertheless, its use remains limited, with only a minority of surgeons reporting its application. Notably, adoption was more prevalent in settings with MDTs. Rupp et al. demonstrated that local antibiotic therapy utilization increased following multidisciplinary interventions.^12^ Among those who applied local therapy, PMMA was the most frequently used carrier, consistent with previous studies showing favorable outcomes with antibiotic-loaded cement beads in managing severe open fractures.^38^

Risk stratification for infection in elderly patients with intertrochanteric fractures to determine the duration of surgical prophylaxis is practiced routinely by only a small proportion of surgeons, though more frequently in multidisciplinary care settings. Elderly patients are particularly susceptible to infection due to immunosenescence and colonization by MDR pathogens, often a consequence of prior healthcare exposure or residency in long-term care facilities. According to previous publication, these patients likely require individualized antimicrobial strategies.^39^ Multidisciplinary approaches in geriatric fracture care have also been associated with reduced mortality and clinical deterioration, as shown in a 2021 Cochrane systematic review.^40^

Timely and appropriate management of the skin and soft tissue envelop, ideally within 72 h, is essential for FRI prevention, especially in high-energy injuries such as Gustilo-Anderson IIIB fractures.^41^ However, our survey indicates that few trauma surgeons routinely collaborate with orthoplastic surgeons, though this is more common in settings with infectious disease specialists. Kotsougiani-Fischer et al. demonstrated that MDT ‒ including trauma, plastic, and vascular surgeons - achieved high success rates in managing complex limb defects.^16^ Additionally, many Brazilian trauma surgeons reported using negative pressure wound therapy to optimize the wound bed prior to definitive closure, particularly in services with MDT involvement. While this technique is widely adopted, evidence supporting its role in FRI prevention remains limited and inconclusive, warranting individualized clinical application.^42^

Regarding current diagnostic strategies towards FRIs, survey responses reflected a predominant reliance on clinical superficial sign and symptoms of infection, blood tests, and radiographic imaging. However, these aspects have lost power according to the current international consensus criteria for FRI diagnosis.^18^^,^^43^ Recommended microbiological diagnostic methods, such as culture-based identification including retrieved-implant sonication, were less frequently reported, whereas histopathological examination was rarely utilized. Biddle et al. found that implementing a multidisciplinary approach in managing periprosthetic joint infections improved access to microbiological diagnostics, facilitating appropriate therapy.^44^ Our previous findings support this, as trauma surgeons working within MDTs are more likely to use sonication fluid to enhance diagnostic accuracy.^45^

This study presents several limitations inherent to survey-based, cross-sectional research. First, the relatively low response rate (24 %) may affect the generalizability of our findings. While this level of participation is consistent with similar international surveys targeting orthopaedic trauma surgeons using comparable dissemination strategies,^17^^,^^38^ a longer data-collection period, additional reminders, and post-stratification weighting based on region or institution type could have enhanced representativeness. Additionally, the potential for non-response bias cannot be excluded, particularly given that most respondents were from the Southeast region of Brazil, home to the country’s largest trauma centers and more structured healthcare systems, which may have skewed results toward more favorable clinical practices. Despite these limitations, our sample included a balanced representation from both public and private institutions, and our analytical focus on the presence or absence of MDTs was grounded in growing evidence that such collaboration improves management of complex musculoskeletal infections.^12^ The study also did not include a formal sample size calculation or power analysis, as it was exploratory in its nature and aimed to describe national practices rather than test predefined hypotheses. Although this approach is supported by methodological guidance for clinician surveys,^22^ we acknowledge that limited precision may constrain the interpretability of certain estimates. While we used a Mini Delphi approach to design the questionnaire, this method has intrinsic constraints, such as limited depth of expert interaction and potential for superficial consensus.^46^ Another limitation is the absence of patient-level outcomes or infection rates, limiting the ability to draw causal inferences for clinical efficacy of reported practices. Lastly, the study did not directly evaluate the economic impact of MDT implementation or guideline-based care pathways.^47^ Any mention of potential cost reductions is speculative and based on previously published evidence suggesting that multidisciplinary strategies may optimize resource use and reduce complication-related expenditures.^48^

This national survey provides the first comprehensive insight into current FRI-related practices among Brazilian trauma surgeons. The findings indicate that consistent collaboration with well-experienced MDTs results in more structured preventive and diagnostic approaches. These results highlight the importance of establishing standardized national protocols that integrate infectious disease specialists and other key professionals into the care of musculoskeletal infections. Future studies employing prospective designs, with larger sample sizes, formal hypothesis testing, and economic evaluations, are warranted to validate our findings and inform evidence-based policies aimed at improving patient outcomes and reducing the burden of fracture-related infections within the Brazilian healthcare system.

## Authors’ contribution

I.S.O., F.B.R., and M.J.S. conceptualized the study’s objectives. I.S.O., M.J.S., F.B.R., C.A.F., T.S.D., G.B.K. and C.C.R. participated in the design of the survey. I.S.O., T.C.R., C.C.R., R.P.V., C.A.F., and T.S.D. disseminated the survey instrument to trauma surgeons. F.B.R. facilitated access to trauma surgeons affiliated with SBOT. I.S.O. and M.J.S. performed data analysis, and R.B.T. contributed to data interpretation. All authors critically reviewed the manuscript, and the sequence of author names was mutually agreed upon.

Correspondence and requests for materials should be addressed to M.J.S.

## Additional information

All research was performed in accordance with relevant guidelines/regulations. Ethical approval for this study was obtained from the Research Ethics Committee of the Federal University of São Paulo – UNIFESP/Hospital São Paulo (Process Number: 6.095.500). Informed consent was obtained from all patients included in the study. This study was performed in accordance with the Declaration of Helsinki.

## Funding

This research is part of a larger project entitled “Epidemiological and microbiological analysis of infections associated with orthopedic fractures in Brazil: An observational, multicenter cohort study”, funded by 10.13039/501100003593CNPq under process number 402,516/2021–4. However, in this part, no specific funding was received from public, commercial, or non-profit sector funding agencies.

## Conflicts of interest

The authors declare no conflicts of interest.

## Appendix Supplementary materials

Image, application 1

## References

[1] Moriarty T.F., Metsemakers W.J., Morgenstern M., Hofstee M.I., Vallejo Diaz A., Cassat J.E. (2022). Fracture-related infection. Nat Rev Dis Primers.

[2] Papakostidis C., Kanakaris N.K., Pretel J., Faour O., Morell D.J., Giannoudis P.V. (2011). Prevalence of complications of open tibial shaft fractures stratified as per the Gustilo-Anderson classification. Injury.

[3] McQuillan T.J., Cai L.Z., Corcoran-Schwartz I., Weiser T.G., Forrester J.D. (2018). Surgical site infections after open reduction internal fixation for trauma in low and middle human development index countries: a systematic review. Surg Infect (Larchmt).

[4] Brasil. Ministério da Saúde. Sistema De Informações sobre Mortalidade. Sistema de Internações Hospitalares [Internet]. Brasília: Ministério da Saúde; [cited 2023 Mar 22]. Available from: https://datasus.saude.gov.br/informacoes-de-saude-tabnet/.

[5] World Health Organization (2022). https://www.who.int/health-topics/road-safety#tab=tab_1.

[6] Brasil. Instituto de Pesquisa Econômica Aplicada (2020). https://www.ipea.gov.br.

[7] Prebianchi S., Santos E.C., Dell’Aquila A., Finelli C., Reis F.B., Salles M.J. (2022). Type of antibiotic but not the duration of prophylaxis correlates with rates of fracture-related infection. Eur J Orthop Surg Traumatol.

[8] Jorge L.S., Fucuta P.S., Gonçalves M.G., Nakazone M.A., de Jesus A, Chueire A.G. (2018). Outcomes and risk factors for polymicrobial posttraumatic osteomyelitis. J Bone Jt Infect.

[9] Woffenden H., Yasen Z., Burden E., Douthwaite A., Elcock S.B., McLean L. (2023). Fracture-related infection: analysis of healthcare utilisation and associated costs. Injury.

[10] Vasoo S., Chan M., Sendi P., Berbari E. (2019). The value of Ortho-ID teams in treating bone and joint infections. J Bone Jt Infect.

[11] Metsemakers W.J., Onsea J., Neutjens E., Steffens E., Schuermans A., McNally M. (2017). Prevention of fracture-related infection: a multidisciplinary care package. Int Orthop.

[12] Rupp M., Walter N., Popp D., Hitzenbichler F., Heyd R., Geis S. (2023). Multidisciplinary treatment of fracture-related infection has a positive impact on clinical outcome: a retrospective case control study at a tertiary referral center. Antibiotics (Basel).

[13] Feihl S., Querbach C., Hapfelmeier A., Busch D.H., von Eisenhart-Rothe R., Gebhardt F. (2022). Effect of an intensified antibiotic stewardship program at an orthopedic surgery department. Surg Infect (Larchmt).

[14] Ntalos D., Berger-Groch J., Rohde H., Grossterlinden L.G., Both A., Luebke A. (2019). Implementation of a multidisciplinary infections conference affects the treatment plan in prosthetic joint infections of the hip: a retrospective study. Arch Orthop Trauma Surg.

[15] Ntalos D., Schoof B., Thiesen D.M., Viezens L., Kleinertz H., Rohde H. (2021). Implementation of a multidisciplinary infections conference improves the treatment of spondylodiscitis. Sci Rep.

[16] Kotsougiani-Fischer D., Fischer S., Warszawski J., Gruetzner P.A., Reiter G., Hirche C. (2021). Multidisciplinary team meetings for patients with complex extremity defects: a retrospective analysis of treatment recommendations and prognostic factors for non-implementation. BMC Surg.

[17] Morgenstern M., Moriarty T.F., Kuehl R., Richards R.G., McNally M.A., Verhofstad M.H.J. (2018). International survey among orthopaedic trauma surgeons: lack of a definition of fracture-related infection. Injury.

[18] Metsemakers W.J., Morgenstern M., McNally M.A., Moriarty T.F., McFadyen I., Scarborough M. (2018). Fracture-related infection: a consensus on definition from an international expert group. Injury.

[19] Govaert G.A.M., Kuehl R., Atkins B.L., Trampuz A., Morgenstern M., Obremskey W.T. (2020). Diagnosing fracture-related infection: current concepts and recommendations. J Orthop Trauma.

[20] Sousa R., Carvalho A., Santos A.C., Abreu M.A. (2021). Optimal microbiological sampling for the diagnosis of osteoarticular infection. EFORT Open Rev.

[21] Rupp M., Walter N., Brochhausen C., Alt V. (2024). Fracture related infection: challenges in definition and diagnosis. J Orthop.

[22] Burns K.E.A., Duffett M., Kho M.E., Meade M.O., Adhikari N.K.J., Sinuff T. (2008). A guide for the design and conduct of self-administered surveys of clinicians. CMAJ.

[23] Shelley A., Horner K. (2021). Questionnaire surveys – sources of error and implications for design, reporting and appraisal. Br Dent J.

[24] Goodfellow L.T. (2023). An overview of survey research. Respir Care.

[25] Marques J.B.V., Freitas D. (2018). Método Delphi: caracterização e potencialidades na pesquisa em educação. Pro-Posições.

[26] Jones T.L., Baxter M.A.J., Khanduja V. (2013). A quick guide to survey research. Ann R Coll Surg Engl.

[27] Jebb A.T., Ng V., Tay L. (2021). A review of key Likert scale development advances: 1995–2019. Front Psychol.

[28] Hammer M.J. (2017). Ethical considerations for data collection using surveys. Oncol Nurs Forum.

[29] Zimba O., Gasparyan A.Y. (2023). Designing, conducting, and reporting survey studies: a primer for researchers. J Korean Med Sci.

[30] Hanssen J.L.J., van der Linden H.M.J., van der Beek M.T., van der Wal R.J.P., Termaat M.F., de Boer M.G.J. (2025). Implementation of multidisciplinary team decisions on the management of complex bone and joint infections: an observational study. BMC Musculoskelet Disord.

[31] Chang Y., Bhandari M., Zhu K.L., Mirza R.D., Ren M., Kennedy S.A. (2019). Antibiotic prophylaxis in the management of open fractures. JBJS Rev.

[32] Vidal P., Fourniols E., Junot H., Meloni C., Bleibtreu A., Aubry A. (2022). Antibiotic stewardship in treatment of osteoarticular infections based on local epidemiology and bacterial growth times. Microbiol Spectr.

[33] Vanvelk N., Chen B., van Lieshout E.M.M., Zalavras C., Moriarty T.F., Obremskey W.T. (2022). Duration of perioperative antibiotic prophylaxis in open fractures: a systematic review and critical appraisal. Antibiotics (Basel).

[34] Bauer S., Bouldouyre M.A., Oufella A., Palmari P., Bakir R., Fabreguettes A. (2012). Impact of a multidisciplinary staff meeting on the quality of antibiotherapy prescription for bone and joint infections in orthopedic surgery. Med Mal Infect.

[35] Royere A.E., Pourrat X., Le Nail L.R., Lartigue M.F., Lemaignen A., Tuloup V. (2024). Impact of pharmacist-led interventions in a multidisciplinary consultation meeting for bone and joint infection. Infect Dis Now.

[36] Vargas-Hernández J.S., Sánchez C.A., Renza S., Leal J.A. (2023). Effectiveness of antibiotic-coated intramedullary nails for open tibia fracture infection prevention: a systematic review and meta-analysis. Injury.

[37] O'Toole R.V., Joshi M., Carlini A.R., Murray C.K., Allen L.E., Huang Y. (2021). Effect of intrawound vancomycin powder in operatively treated high-risk tibia fractures. JAMA Surg.

[38] Puetzler J., Zalavras C., Moriarty T.F., Verhofstad M.H.J., Kates S.L., Raschke M.J. (2019). Clinical practice in prevention of fracture-related infection: an international survey among 1197 orthopaedic trauma surgeons. Injury.

[39] Rodríguez-Pardo D., Escolà-Vergé L., Sellarès-Nadal J., Corona P.S., Almirante B., Pigrau C. (2021). Periprosthetic joint infection prophylaxis in the elderly after hip hemiarthroplasty in proximal femur fractures: insights and challenges. Antibiotics (Basel).

[40] Handoll H.H., Cameron I.D., Mak J.C., Panagoda C.E., Finnegan T.P. (2021). Multidisciplinary rehabilitation for older people with hip fractures. Cochrane Database Syst Rev.

[41] Luo J., Zhou M., Lin F., Wang J., Rui Y. (2023). Clinical effectiveness of early internal fixation combined with free flap technique in the treatment of Gustilo IIIB open forearm fracture. Orthop Traumatol Surg Res.

[42] Liu X., Zhang H., Cen S., Huang F. (2018). Negative pressure wound therapy versus conventional wound dressings in treatment of open fractures: a systematic review and meta-analysis. Int J Surg.

[43] Alt V., McNally M., Wouthuyzen-Bakker M., Metsemakers W.J., Marais L., Zalavras C. (2024). The FRI classification: a new classification of fracture-related infections. Injury.

[44] Biddle M., Kennedy I.W., Wright P.M., Ritchie N.D., Meek R.M.D., Rooney B.P. (2021). Improving outcomes in acute and chronic periprosthetic hip and knee joint infection with a multidisciplinary approach. Bone Joint Open.

[45] Ribeiro T.C., Honda E.K., Daniachi D., Cury R.P.L., da Silva C.B., Klautau G.B. (2021). The impact of sonication cultures when the diagnosis of prosthetic joint infection is inconclusive. PLoS One.

[46] Filho F.J. (2011).

[47] Lima A.L.L., Oliveira P.R., Carvalho V.C., Cimerman S., Savio E. (2014). Recommendations for the treatment of osteomyelitis. Braz J Infect Dis.

[48] Nyffeler R., Morgenstern M., Osinga R., Kuehl R., Gahl B., Imhof A. (2025). Fracture-related infections of the lower extremity: analysis of costs and their drivers. Injury.

