# Supplementary material for: The impact of a multidisciplinary team on the management of fracture-related infections: A surveillance study in Brazil
Source: Braz J Infect Dis. 2025 Sep 11;29(6):104576. doi: 10.1016/j.bjid.2025.104576 (PMC12793792; doi:10.1016/j.bjid.2025.104576)
Supplement: Supplementary file 1 [file mmc1.docx]

**Appendix A**

**Questions**

1. Are the infection prevention protocols adopted in fracture management in Brazil homogeneous? What measures do you consider that most surgeons adopt or do not adopt uniformly?
2. What parameters do you use to indicate combined surgical antibiotic prophylaxis after closed and open fractures?
3. What parameters do you use to extend antibiotic prophylaxis during hospitalization for closed and open fractures?
4. What parameters do you use to indicate antibiotic therapy after hospital discharge in the treatment of closed and open fractures?
5. What parameters do you use to indicate local and systemic antibiotic therapy in the management of closed and open fractures?
6. Regarding local antibiotic therapy in fractures, do you use powdered or liquid antibiotics, or do you combine them with biomaterials?
7. If biomaterials are used, which one do you use most frequently in local therapy?
8. What parameters do you use to indicate surgical treatment in one or two stages (e.g., external fixator) for closed and open fractures?
9. What clinical, laboratory, and/or radiological markers are essential in developing the diagnostic algorithm for fracture-related infection?
10. What is the impact of the presence of a multidisciplinary team on the development of prevention, diagnosis, and treatment methods for fracture-related infection? And who should compose this team?

**Appendix B**

**Preventive Strategies and Diagnostic Methods for Fracture-Related Infections**

Dear Surgeon,

We would like to invite you to complete the first questionnaire aimed at understanding the preventive strategies and diagnostic methods for fracture-related infections in Brazil.

Your participation is very important to us. Please take a moment to respond—it will only take 10 minutes.

Below, we provide the informed consent form.

Date:

Below is the Informed Consent Term (IC) available. Do you agree?

Access to the IC:[Attachment: "IC_PREVENTIVE_STRATEGIES_AND_DIAGNOSTIC_METHODS_FI.doc"]

Participant's name:

Do you agree?

**Demographic Aspects**

1. In which region of Brazil do you work?

- North
- Northeast
- Midwest
- Southeast
- South

1. What is the type of hospital where you most frequently practice?

- Academic
- Public
- Philanthropic
- Private
- Mixed

1. How many years of experience do you have in orthopedics?

- 0-5 years
- 5-10 years
- 10-20 years
- 20 years

**Strategies for fracture-related infection prevention**

**Multidisciplinary team**

1. In the service where you most frequently practice, is there collaboration with the multidisciplinary team to establish measures for prevention, diagnosis, and treatment of FRIs?

- Always
- Frequently
- Occasionally
- Rarely
- Never

**Irrigation and debridament**

1. In the service where you most frequently practice, what type of solution is used for the irrigation of open fractures?

- Normal saline
- Normal saline + antibiotics
- Normal saline + antiseptics
- Antiseptics
- Distilled water
- Others
  If others, please describe:

1. In the service where you most frequently practice, is the irrigation of open fractures performed with any high-pressure instrument?

- No pressure in the irrigation technique
- Yes, with low pressure
- Yes, with high pressure

1. In the service where you most frequently practice, what is the volume of solution used for the irrigation of GI and/or GII open fractures?

- < 3L
- 3 to 6L
- 7 to 9L
- > 9L

1. In the service where you most frequently practice, what is the volume of solution used for the irrigation of GIII open fractures?

- < 3L
- 3 to 6L
- 7 to 9L
- > 9L

1. In the service where you most frequently practice, what is the average time elapsed from the patient's admission to the emergency room with a GI and/or GII open fracture to debridement and fracture stabilization?

- < 6h
- 6 to 24h
- 24 to 48h
- > 48h

1. In the service where you most frequently practice, what is the average time elapsed from the patient's admission to the emergency room with a GIII open fracture to debridement and fracture stabilization?

- < 6h
- 6 to 24h
- 24 to 48h
- > 48h

**Surgical prophylaxis**

1. In the service where you most frequently practice, is the dose of prophylactic antibiotic adjusted based on the patient's approximate weight?

- Always
- Frequently
- Occasionally
- Rarely
- Never

1. In the service where you most frequently operate, what is the average time elapsed from the patient's admission with a GI and/or GII open fracture to the administration of prophylactic antibiotic?

- 3h
- 3-6h
- 7-12h
- 13-24h

1. In the service where you most frequently operate, what is the average time elapsed from the patient's admission with a GIII open fracture to the administration of prophylactic antibiotic?

- 3h
- 3-6h
- 7-12h
- 13-24h

1. Which antibiotic(s) are used for prophylaxis in the surgery for closed fracture correction? (You can select more than one option)

- Cephalosporins (e.g., Cefazolin, Cefalexin, Cefuroxime)
- Aminoglycosides (e.g., Gentamicin)
- Glycopeptides (e.g., Vancomycin, Teicoplanin)
- None of these
- Other(s) (e.g., Ciprofloxacin, Clindamycin)

If others, please describe:

1. Which antibiotic(s) are used for prophylaxis in the surgery for GI or GII open fracture correction? (You can select more than one option)

- Cephalosporins (e.g., Cefazolin, Cefalexin, Cefuroxime)
- Aminoglycosides (e.g., Gentamicin)
- Glycopeptides (e.g., Vancomycin)
- None of these
- Other(s) (e.g., Ciprofloxacin, Clindamycin)

If others, please describe:

1. Which antibiotic(s) are used for prophylaxis in the surgery for GIII open fracture correction? (You can select more than one option)

- Cephalosporins (e.g., Cefazolin, Cefalexin, Cefuroxime)
- Aminoglycosides (e.g., Gentamicin)
- Glycopeptides (e.g., Vancomycin)
- None of these
- Other(s) (e.g., Ciprofloxacin, Clindamycin)

If others, please describe:

1. For closed fractures, for how many days is the prophylactic antibiotic maintained in the postoperative period?

- 1 day
- 2 days
- 3 days
- 4 days
- 5 days
- > 5 days

1. For GI and/or GII open fractures, how many days is the prophylactic antibiotic maintained in the postoperative period?

- 1 day
- 2 days
- 3 days
- 4 days
- 5 days
- > 5 days

1. For GIII open fractures, how many days is the prophylactic antibiotic maintained in the postoperative period?

- 1 day
- 2 days
- 3 days
- 4 days
- 5 days
- > 5 days

1. In the management of GI or GII open fractures, is local antibiotic therapy (e.g., cement [PMMA] with antibiotic) used?

- Always
- Frequently
- Occasionally
- Rarely
- Never

1. In the management of GIII open fractures, is adjunctive local antibiotic therapy (e.g., cement [PMMA] with antibiotic) used?

- Always
- Frequently
- Occasionally
- Rarely
- Never

1. In the management of open fractures, is local antibiotic therapy used in combination with any biomaterial/ceramic? (You can select more than one option)

- PMMA
- Calcium sulfate
- Tricalcium phosphate with hydroxyapatite
- Bioglass
- None of these
- Other(s)
  If others, please describe:

1. What dose of antibiotics are used in local therapy (with PMMA or another biomaterial)? (You can select more than one option)

- Vancomycin up to 2g
- Vancomycin above 2g
- Gentamicin up to 240 mg
- Gentamicin above 240 mg
- Other antibiotic with another dose
- I don’t know

If other, please describe the name and dose of the antibiotic:

1. In the service where you most frequently practice, is local antibiotic therapy (e.g., Vancomycin) used without a vehicle (e.g., PMMA) in open fractures?

- Always
- Frequently
- Occasionally
- Rarely
- Never

1. Which antibiotic(s) are used for prophylaxis in the surgery for trochanteric and femoral neck fractures in elderly patients in the service where you most frequently practice? (You can select more than one option)

- Cephalosporins (e.g., Cefazolin, Cefalexin, Cefuroxime)
- Aminoglycosides (e.g., Gentamicin)
- Glycopeptides (e.g., Vancomycin)
- None of these
- Other(s) (e.g., Ciprofloxacin, Clindamycin)

If other, please describe:

1. In the service where you most frequently practice, is there a risk stratification conducted to evaluate the extension of antibiotic prophylaxis in the surgery for trochanteric and femoral neck fracture correction in elderly patients?

- Always
- Frequently
- Occasionally
- Rarely
- Never

1. In the service where you most frequently practice, is the duration of the prophylactic antibiotic used in the surgery for trochanteric and femoral neck fracture correction in elderly patients extended after hospital discharge?

- Yes
- No

1. For closed or open fractures, in the absence of clinical signs of infection, which antibiotic(s) are prescribed at the time of patient discharge? (You can select more than one option)

- Cefalexin
- Ciprofloxacin
- Clindamycin
- Amoxicillin
- No antibiotic
- Other(s)

If other, please specify:

**Management of skin and soft tissues**

1. In the service where you practice, is there a multidisciplinary team that includes a plastic surgeon for the treatment of severe skin and soft tissue injuries related to open fractures?

- Always
- Frequently
- Occasionally
- Rarely
- Never

1. In the management of complicated skin and soft tissue, is any adjuvant therapy used? (You can select more than one option)

- Negative pressure therapy
- Local antibiotics
- Saline solution + local antiseptics
- Other(s)
- None

If others, please specify:

**Diagnosis of FRI**

1. What parameters are used to confirm the diagnosis of fracture-related infection?

- Fever and inflammatory signs at the surgical site (erythema, heat, redness)
- Detection of fistula
- Purulent secretion at the surgical site
- Increase in inflammatory markers (leukogram, CRP, or ESR)
- Microbiological identification of the same pathogen in at least two separate samples collected from deep tissue and/or implant
- Imaging exams (ultrasound, CT, or MRI) showing alterations consistent with infection

1. In the diagnosis of Fracture-Related Infection, from which locations are microbiological samples collected for analysis? (You can select more than one option)

- Soft tissue samples from the surgical site
- Secretions from the surgical wound
- Bone fragments
- Sonication culture of the removed implant
- Swab of intraoperative secretion

1. In suspected FRI diagnosis, how many tissue fragments are collected?

- 1
- 2
- 3
- 4
- 5
- > 5

1. In the service where you practice, is pathological examination of bone tissue samples with suspected osteomyelitis routinely performed?

- Always
- Frequently
- Occasionally
- Rarely
- Never
